# The University Medical Magazine. Professor Reise’s Letters

**Published:** 1891-01

**Authors:** 


The University Medical Magazine announces that, with the beginning of the New Year, it will add a new department to its columns devoted to Medical Progress. It will be divided into five sections, each under charge of special editors, as follows: Medicine, conducted by William Pepper, M. D., and James Tyson, M. D.; Surgery, by D. Hayes Agnew, M. D., and J. William White, M. D.; Therapeutics, by Horatio C. Wood, M. D.; Gynecology, by William Goodell, M. D.; Obstetrics, by Barton Cooke Hirst, M. D. The advantages of this plan are obvious, and are such as every progressive medical magazine must adopt. The *University* expects to make this one of the most attractive features of this already very attractive periodical, and will increase its size by the addition of sixteen to twenty-four pages, to make room for the new feature. It will, however, not advance its subscription price on that account. We congratulate the management of this excellent journal on its success already achieved, and bespeak for it a brilliant future.

---

We devote considerable space in this issue to two letters of Professor Reise, of Copenhagen, (kindly translated for us by Dr. Mynter,) giving his observations of Koch's recent methods of treating tuberculosis. It is by far the best exposition of the subject that we have yet seen, and we trust our readers will be interested in its perusal. We shall present the valuable points on this subject from time to time as they are evolved, but shall avoid filling the Journal with unreliable data. The general status, as expressed in our leader in December, is still unchanged.

---

The District Medical Society of the County of Hudson, N. J., has adopted a set of preambles and resolutions calling upon the Legislature to pass a bill to be introduced by the New Jersey State Board of Medical Examiners, repealing the charter of the "Medical and Surgical College of the State of New Jersey," for alleged irregular practices in methods of teaching and issuing diplomas.

